# Impact of the COVID-19 pandemic on cardiac magnetic resonance imaging practices: insights from the MRCT registry

**DOI:** 10.1007/s00330-025-11464-w

**Published:** 2025-02-19

**Authors:** Lukas J. Moser, Costanza Lisi, Matthias Gutberlet, Sara Boccalini, Ricardo P. J. Budde, Marco Francone, Maja Hrabak Paar, Christian Loewe, Giuseppe Muscogiuri, Luigi Natale, Konstantin Nikolaou, Maja Pirnat, Rodrigo Salgado, Rozemarijn Vliegenthart, Michelle C. Williams, Matthias Eberhard, Hatem Alkadhi

**Affiliations:** 1 Diagnostic and Interventional Radiology, University Hospital Zurich, University of Zurich, Zurich, Switzerland; 2 Department of Biomedical Sciences, Humanitas University, Milan, Italy; 3 IRCCS Humanitas Research Hospital, Milan, Italy; 4 Department of Diagnostic and Interventional Radiology, University of Leipzig—Heart Centre, Leipzig, Germany; 5 Department of Cardiovascular Radiology, Hôpital Pradel, Hospices Civils de Lyon, Lyon, France; 6 Department of Radiology and Nuclear Medicine, Erasmus Medical Center, Rotterdam, The Netherlands; 7 Department of Diagnostic and Interventional Radiology, University Hospital Centre Zagreb, Zagreb, Croatia; 8 Division of Cardiovascular and Interventional Radiology, Department of Biomedical Imaging and Image-Guided Therapy, Medical University Vienna, Vienna, Austria; 9 Department of Radiology, ASST Papa Giovanni XXIII, Bergamo, University Milano Bicocca, Milan, Italy; 10 Department of Radiological Sciences—Institute of Radiology, Catholic University of Rome, A. Gemelli University Hospital, Rome, Italy; 11 Department of Diagnostic and Interventional Radiology, University of Tübingen, Tübingen, Germany; 12 Radiology Department, University Medical Centre Maribor, Maribor, Slovenia; 13 Department of Radiology, Antwerp University Hospital, Edegem, Belgium; 14 Department of Medicine & Health Sciences, University of Antwerp, Wilrijk, Belgium; 15 Department of Radiology, Holy Heart Hospital, Lier, Belgium; 16 Department of Radiology, University of Groningen, University Medical Center Groningen, Groningen, The Netherlands; 17 British Heart Foundation Centre of Research Excellence, University of Edinburgh, Edinburgh, UK

**Keywords:** Myocarditis, Registries, COVID-19, Magnetic resonance imaging, Heart

## Abstract

**Purpose:**

To assess the relationship between the COVID-19 pandemic and the spectrum of indications for cardiac magnetic resonance imaging (MRI) with a focus on myocarditis.

**Materials and methods:**

This was a retrospective analysis of data from the MRCT registry of the European Society of Cardiovascular Radiology (ESCR). Data regarding indications and diagnoses of myocarditis between January 1, 2018, and April 30, 2024, were extracted. Data was analyzed for the periods before and after the beginning (i.e., March 11, 2020) of the COVID-19 pandemic.

**Results:**

Data from 112,361 cardiac MRI examinations was analyzed (63.5% male, median age 58 [IQR 44–69]). Over the entire period, assessment of myocarditis was the most common indication for cardiac MRI (31%, *n* = 34,906/112,361). Before the pandemic, this indication comprised 28% of examinations and increased to a maximum of 41% in 2022 after the onset of the pandemic. Simultaneously, the positivity rate of these examinations decreased from 21% before the pandemic to 14% in 2022. Male patients had a higher positivity rate than female patients both before and during the pandemic, with mirroring trends between sexes. The proportion of cardiac MRI examinations performed for suspected and known coronary artery disease showed an inverse relationship with those performed for myocarditis and decreased from 24% and 17% pre-pandemic to a minimum of 21% and 13% during the pandemic.

**Conclusion:**

The COVID-19 pandemic considerably influenced the pattern of referrals for cardiac MRI examinations in Europe, leading to a higher proportion of examinations for suspected myocarditis but a reduced positivity rate, suggesting a lower referral threshold for this indication. At the same time, proportionally fewer examinations were performed for suspected and known coronary artery disease.

**Key Points:**

***Question*** *The COVID-19 pandemic may have influenced the spectrum and positivity rates of indications for cardiac MRI, especially examinations for myocarditis*.

***Findings*** *The COVID-19 pandemic led to a higher proportion of cardiac MRI examinations for suspected myocarditis but a reduced positivity rate*.

***Clinical relevance*** *The spectrum and proportions of indications for cardiac MRI give important information on the historical and current trends in cardiac imaging and provide insight into resource deployment*.

## Introduction

On March 11, 2020, the World Health Organization (WHO) declared the outbreak of the severe acute respiratory syndrome coronavirus 2 (SARS-CoV-2) a pandemic [1]. The SARS-CoV-2-induced disease COVID-19 has since claimed the lives of 7,071,324 people worldwide as of October 13, 2024 [2]. The declaration led to the decree of large-scale lockdowns restricting contact between people in large parts of the world to reduce the spread of infection [3]. The pandemic also greatly influenced the medical community, putting triaging systems in place [4, 5], postponing elective procedures [6], implementing hygiene and isolation protocols [7, 8], and also leading to a shortage of personal protection equipment [9]. The INCAPS (International Atomic Energy Agency Noninvasive Cardiology Protocols Study) COVID surveys demonstrated that cardiac imaging was no exception to these effects and found a significant drop in examination numbers in March and April 2020 when compared to one year before the pandemic [10, 11], with varying recovery rates across countries one year later [12]. These studies however only reported on the total number of examinations and did not investigate in detail the specific indications for the various imaging modalities.

Early after the SARS-CoV-2 outbreak, reports emerged that infection carried an increased risk of acute cardiovascular injury such as myocarditis [13–16]. In expedited clinical trials, vaccinations were developed which resulted in a marked reduction in COVID-19 infections [17, 18]. The first vaccinations received emergency use authorization in the UK on December 2, 2020, with the US and Canadian drug authorities following suit, and finally were granted conditional marketing authorization in the European Union on December 21, 2020 [19]. After the start of widespread vaccination campaigns, however, case reports of potential side effects such as myocarditis emerged [20]. While the exact mechanism of this association remains a topic of ongoing research, a similar pathogenic mechanism was suggested for virus-associated myocarditis [21].

The purpose of our study was to assess the relationship of the COVID-19 pandemic with the spectrum of indications for cardiac MRI in Europe, given that cardiac MRI represents the gold standard noninvasive modality for the diagnosis of myocarditis [22, 23]. We used data from the multicenter, multi-national MRCT registry representing one of the largest databases of cardiac MRI examinations to address the impact of COVID-19 on the indications of cardiac MRI, which to our knowledge has not yet been investigated. Our hypothesis was that the COVID-19 pandemic led to a relative increase in cardiac MRI examinations for suspected myocarditis along with a change in positivity rate.

## Materials and methods

This retrospective study had ethics committee approval (Leipzig University; no. 131/17-ek). Patient consent was waived due to analyses of anonymized data.

### The MRCT registry

The MRCT registry of the European Society of Cardiovascular Radiology (ESCR) for cardiovascular CT and MRI examinations (www.mrct-registry.org) was established in 2011 to provide a platform for national and international certification of cardiac imagers and imaging centers. The registry provides a centralized database to enter anonymized data from cardiac CT and MRI examinations and allows for the assessment of safety, quality, indications, diagnoses, use of contrast media, scan protocols, and extracardiac findings, among others [24–29].

### Data included in the analysis

Indications and diagnoses for cardiac MRI were extracted from the database spanning from January 01, 2018, to April 30, 2024, including data from around two years before the SARS-CoV-2 outbreak and the COVID-19 pandemic itself. A comparable time interval before and during the pandemic was chosen to mitigate the effects caused by guidelines, recommendations, and local practice changes across institutions and countries.

### Definition of timepoints

The beginning of the COVID-19 pandemic was defined as March 11, 2020, as declared by the WHO [1]. The timespan between January 01, 2018, and March 11, 2020, is considered pre-pandemic. As representative samples of COVID-19 vaccines, the three most frequently used products in the European Union (Comirnaty, Pfizer/BioNTech; Spikevax, Moderna Biotech; Vaxzevria, AstraZeneca) were chosen [30]. Their dates of conditional marketing authorization granted by the European Commission and European Medicines Agency were used as a reference for the start of the vaccination campaign (Comirnaty: December 21, 2020; Spikevax: January 06, 2021; Vaxzevria: January 29, 2021) [31]. The WHO declared the end of the *Public Health Emergency of International Concern* on May 05, 2023, while noting that the pandemic was still ongoing [32].

### Statistical analysis

Descriptive statistical approaches were used to describe the registry data. To summarize data in data periods, dates were rounded to the nearest specified time point. Positive diagnoses for myocarditis were defined as the sum of reported “acute” and “chronic” myocarditis cases. For the calculation of the positivity rate for myocarditis, the positive diagnoses were divided by the number of examinations performed for suspicion of myocarditis. The indication “assessment of myocarditis” was defined as the sum of examinations performed for suspected myocarditis and known myocarditis. Trends in the five most common indications present in this analysis and in a previous publication were analyzed [28] and were analyzed as proportions to avoid bias from fluctuation in absolute yearly registry entries. All proportions are relative to the total number of examinations in a period. Temporal trends in the data are visualized by *LOESS* (Locally Estimated Scatterplot Smoothing) functions and depicted with 95% confidence intervals. For visualization, data was aggregated semi-annually to provide more granular data points for the trendlines. In the results section data was aggregated annually for a more concise presentation. The Chi-square test was applied to compare sex differences and differences in risk stratification between pre-pandemic and pandemic groups. Age, height, body weight, and differences in body mass index (BMI) between groups were assessed by unpaired Student *t*-tests. All analyses were performed in R (version 4.3.1, The R Foundation).

## Results

### Registry baseline data

A total of 112,361 cardiac MRI examinations were included in the MRCT registry between January 1, 2018, and April 30, 2024. Thirty-six countries are registered for the MRCT registry, with the majority of cases being entered by European countries (see Supplementary Table S1). The baseline characteristics of patients are provided in Table 1. No significant differences were found between the pre-pandemic and the pandemic cohort regarding age (*p* = 0.70), height (*p* = 0.23), body weight (*p* = 0.38), and BMI (*p* = 0.21). Differences were found between cohorts in regard to the risk for coronary artery disease and sex (both, *p* < 0.001).

**Table 1:** Baseline characteristics of the patients in the MRCT registry in the investigated pre-pandemic cohort from January 1, 2018, to March 10, 2020, and pandemic cohort from March 11, 2020, to April 30, 2024

Characteristic	Pre-pandemic	Pandemic	*p*-value
Age [years]	58 (43–69)	58 (44–69)	0.70
Sex [male]	38,013 (62.9%)	33,311 (64.2%)	< 0.001
Sex [female]	22,434 (37.1%)	18,603 (35.8%)	
Height [cm]	174 (167–180)	173 (166–180)	0.23
Weight [kg]	80 (69–92)	80 (70–93)	0.38
Body mass index [kg/m^2^]	26.5 (23.5–29.7)	26.1 (23.8–30.1)	0.21
Risk for coronary artery disease^1^	Low 11.9% Medium 82.2% High 5.9%	Low 13.5% Medium 80.0% High 6.5%	< 0.001

Data are median and quartiles in parentheses, or number of patients and percentages in parentheses. ^1^ As defined by Taylor et al [40]

### Indications for cardiac MRI between 01/2018 and 04/2024

**Fig. 1:**
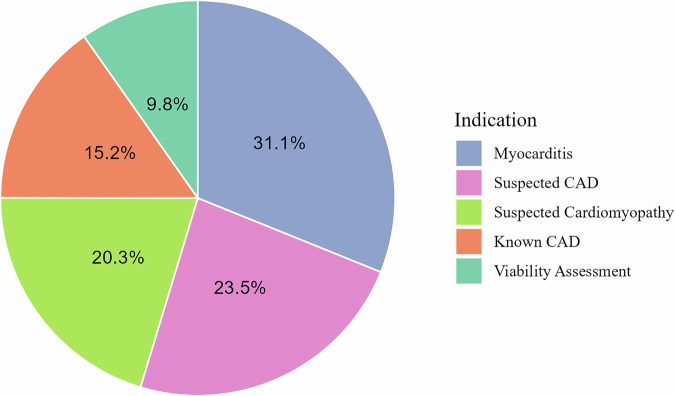
Proportion of the five most common indications for cardiac MRI between January 2018 and May 2024. Note: indications are not mutually exclusive. The proportion in percentage is calculated in relation to the total number of examinations, including indications not listed

Overall, the most common indications for cardiac MRI were diagnosis/rule out of myocarditis (31.1%, *n* = 34,906/112,361), suspicion of coronary artery disease (23.5%, *n* = 26,455/112,361), suspected cardiomyopathy (20.3%, *n* = 22,811/112,361). These were followed by imaging for known coronary artery disease (15.2%, *n* = 17,073/112,361) and viability assessment (9.8%, *n* = 10,960/112,361) (see Fig. 1), the latter showing overlap with 20.1% of cases imaged for known coronary artery disease also underwent imaging for viability assessment.

### Cardiac MRI for myocarditis

**Fig. 2:**
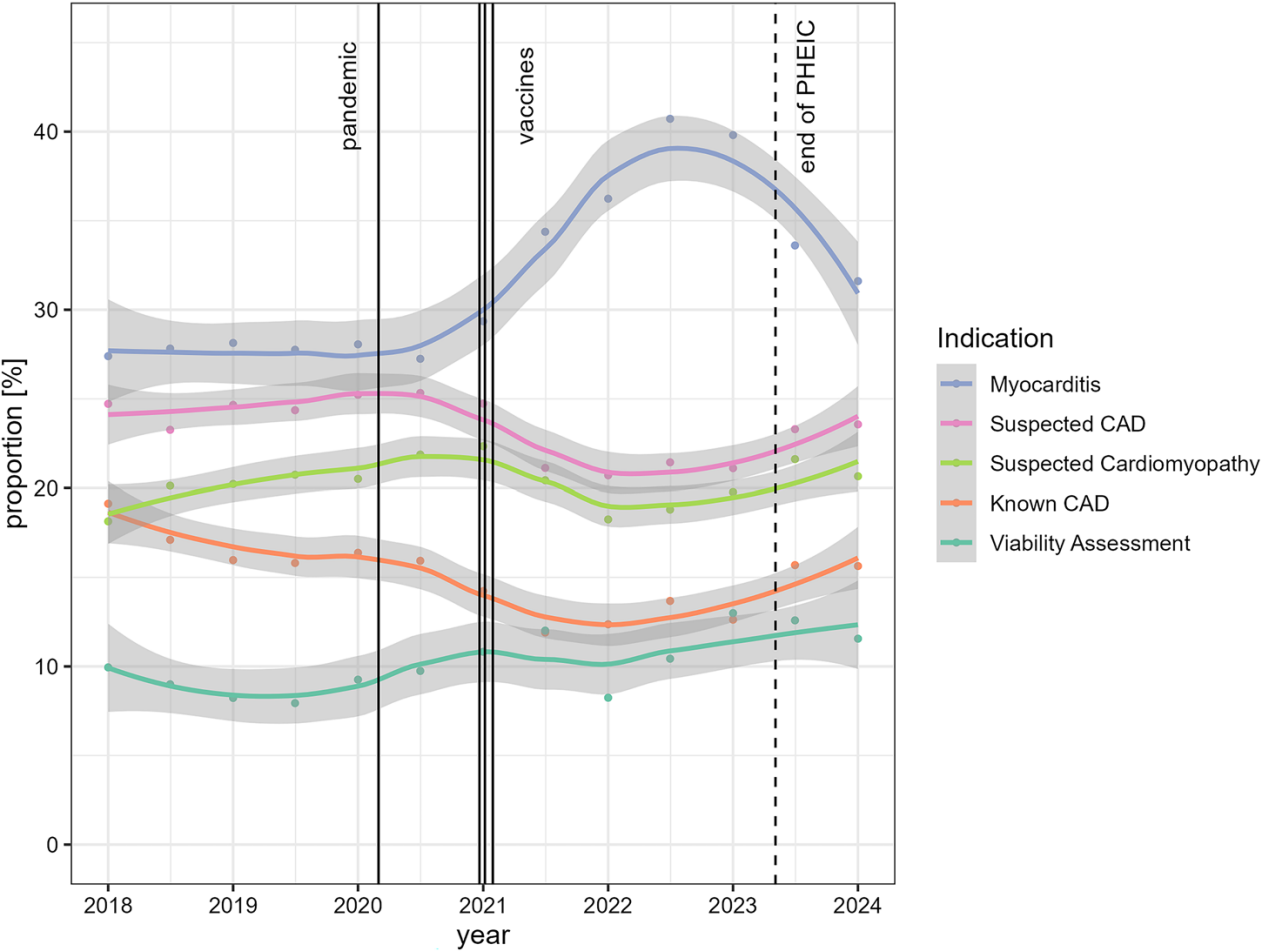
Time course and proportions of the five most common indications in cardiac MRI relative to the total number of cardiac MRI examinations according to the Cardiac ESCR MRCT registry. Vertical lines represent the official beginning of the pandemic, the dates of conditional marketing authorization granted by the European Commission for the three most frequently used vaccines, and the end of the PHEIC. Gray-shaded areas represent 95% confidence intervals derived from the LOESS functions. CAD, coronary artery disease; PHEIC, public health emergency of international concern

From January 1, 2018, until the start of the pandemic (i.e., until March 11, 2020) on average 28.0% of cardiac MRIs were performed for the assessment of myocarditis, which remained at a proportion of 27.7% in 2020, and then markedly increased to 33.2% in 2021 and 40.5% in 2022. The increase began in mid-2020 and accelerated in the following years (Fig. 2).

### Proportion of the remaining most common indications for cardiac MRI

The proportion of examinations for suspected coronary artery disease and known coronary artery disease showed an inverse relationship with those performed for myocarditis and decreased from 24.4% and 16.7% two years pre-pandemic to a minimum of 21.4% and 12.5% in 2022 and 2021, respectively. Examinations for suspected cardiomyopathy and viability assessment fluctuated between 18.6% (2022) and 21.8% (2020) and between 8.1% (2019) and 12.6% (2023), respectively (see Fig. 2).

### Positivity rates of cardiac MRI for the indication of myocarditis

While the relative proportion of cardiac MRI for assessment of myocarditis increased, the proportion of cases that were reported positive for myocarditis declined. From January 2018 to the beginning of the pandemic an average of 20.5% of patients investigated for myocarditis received a positive diagnosis, whereas during the pandemic the positivity rate dropped to 17.8% in 2021 and 14.4% in 2022. Since then, the positivity rate recovered to a lower level than pre-pandemic at 15.2% in 2023 and 18.0% during early 2024.

**Fig. 3:**
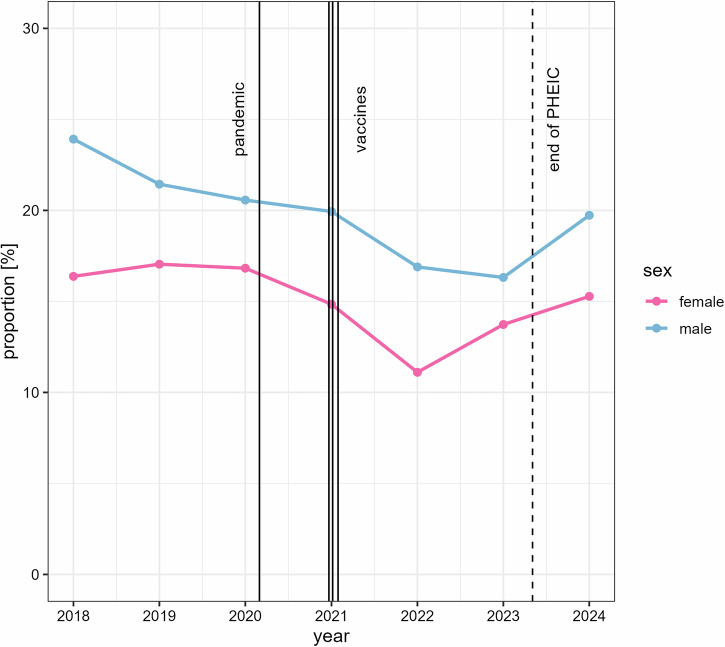
Positivity rate for cardiac MRI performed for myocarditis by year. Vertical lines represent the official beginning of the pandemic, the dates of conditional marketing authorization granted by the European Commission for the three most frequently used vaccines, and the end of the PHEIC. PHEIC, public health emergency of international concern

The positivity rate was higher for male than for female patients in the two years before the pandemic and remained so throughout the observed period. The positivity rate decreased from pre-pandemic (male 23.0%, female 16.8%) to 2021 (male 19.9%, female 14.8%) and 2022 (male 16.9%, female 11.1%), and then recovered in 2023 (male 16.3%, female 13.7%) and early 2024 (male 19.7%, female 15.3%) (Fig. 3).

**Fig. 4:**
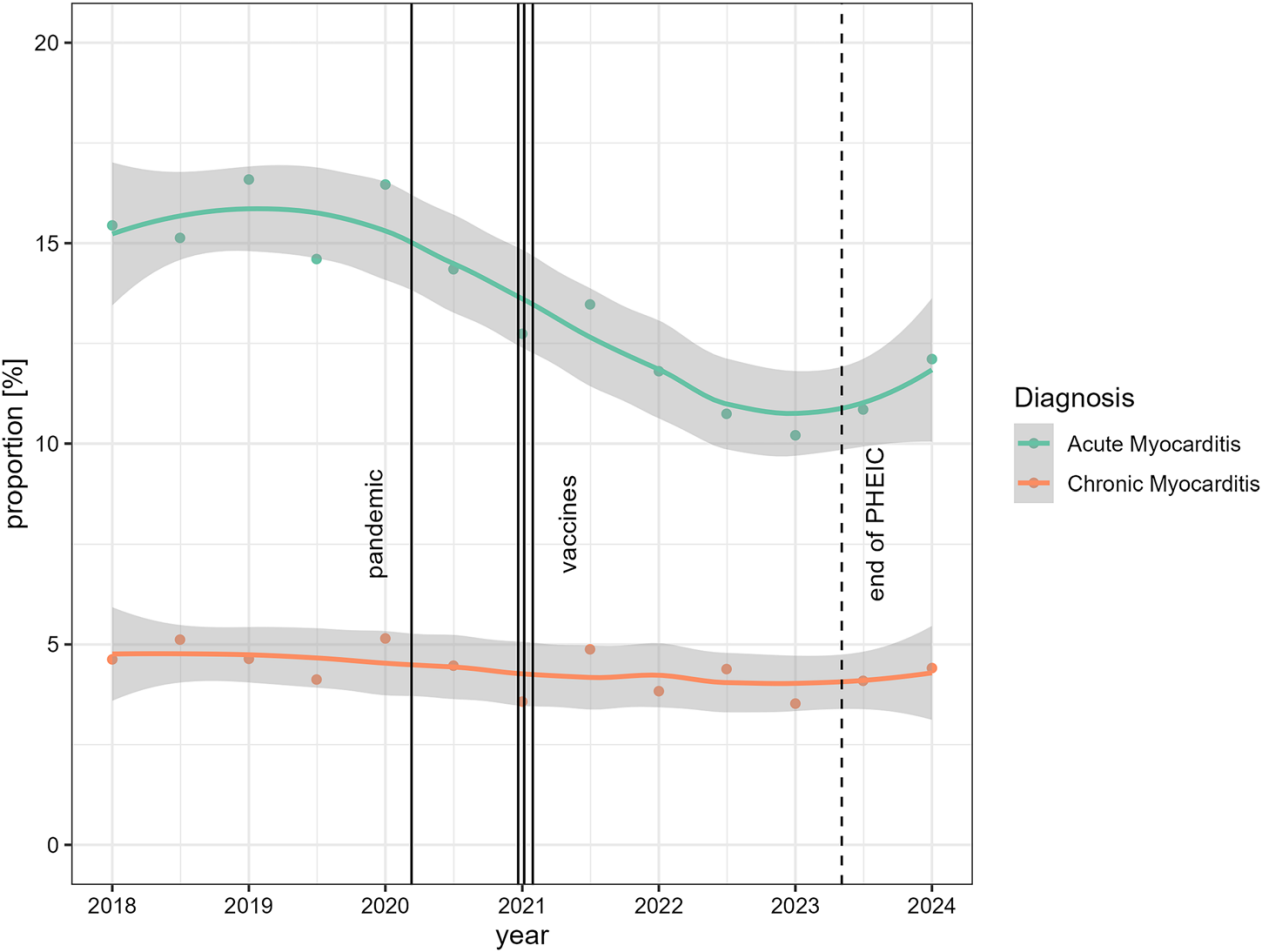
Time course and proportion of cardiac MRI examinations with a positive diagnosis of acute and chronic myocarditis examinations according to the Cardiac ESCR MRCT registry. Vertical lines represent the official beginning of the pandemic, the dates of conditional marketing authorization granted by the European Commission for the three most frequently used vaccines, and the end of the PHEIC. Gray-shaded areas represent 95% confidence intervals derived from the LOESS functions. PHEIC, public health emergency of international concern

It is worth noting that the rate of chronic myocarditis has remained fairly consistent (range 3.6% to 5.6%), indicating that the proportion of acute myocarditis diagnoses was mainly responsible for the observed dynamics (Fig. 4).

### Analysis of sex and age differences in indications and positivity rates of myocarditis

**Fig. 5:**
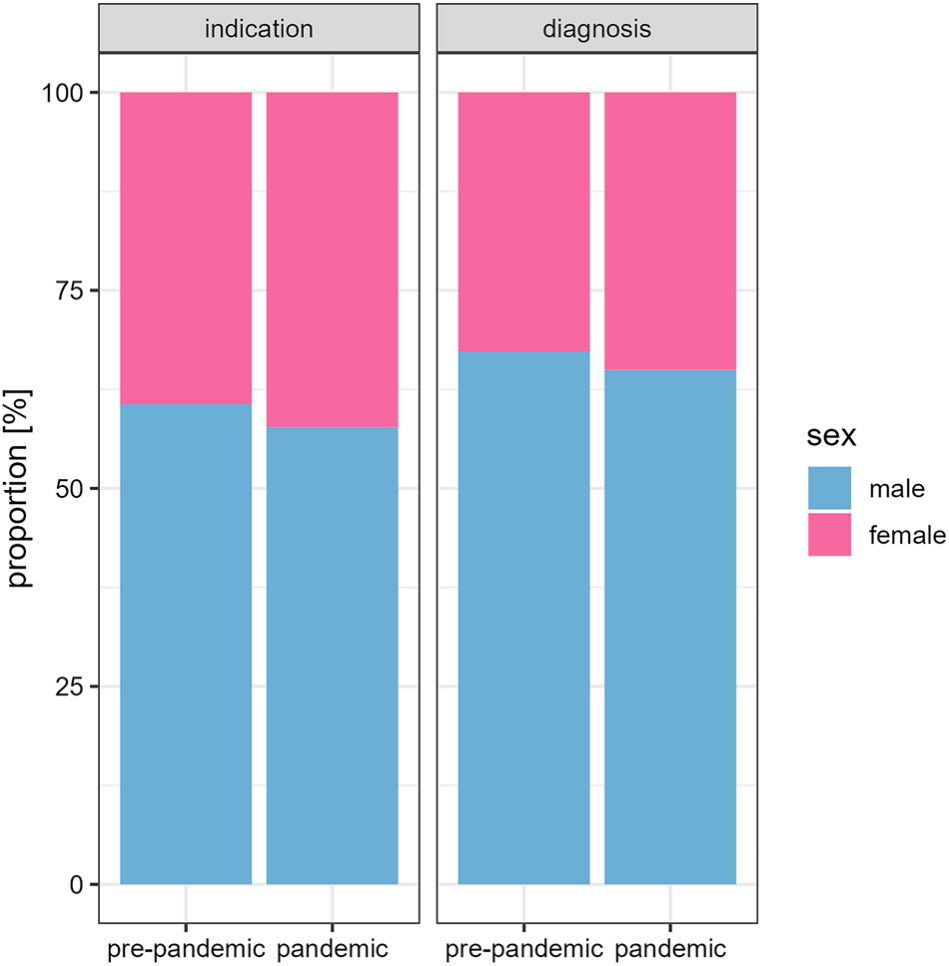
Distribution of sex for the MRI indication of myocarditis and the diagnosis of myocarditis divided in the time before the pandemic on the left and during the pandemic on the right of each pair

MRI examinations for the assessment of myocarditis were generally performed more frequently in men than in women (in the observed period 58.9% of patients were male). In the two years preceding the pandemic, 60.6% of patients examined for myocarditis were male. Since the pandemic started, this fraction decreased significantly to 57.7% (*p* < 0.001) (Fig. 5). In the same intervals, the mean age for patients slightly increased from 47.0 years to 47.5 years (*p* < 0.05).

On average, more male patients received a positive diagnosis for myocarditis (in the observed period 66.1% of patients were male). This fraction significantly decreased from 67.3% pre-pandemic to 65.0% during the pandemic (*p* < 0.05) (see Fig. 5). At the same time, the mean age of patients diagnosed with myocarditis increased slightly from 46.6 years to 47.6 years (*p* < 0.05).

## Discussion

Data from the MRCT registry of the ESCR chronicles the influence of the COVID-19 pandemic on the cardiac MRI landscape by demonstrating: (i) a relative increase in cardiac MRI examinations for the assessment of myocarditis coinciding with the beginning of the pandemic; (ii) a relative decrease in MRI examinations for suspected and known CAD during the same time period; (iii) a decrease in the positivity rate for examinations for suspected myocarditis; (iv) a decrease in the proportion of male and female patients examined for myocarditis and diagnosed with myocarditis; and (v) a slight increase in the mean age of patients examined for myocarditis and diagnosed with myocarditis.

The INCAPS group has investigated the effects of the COVID-19 pandemic on the total number of cardiac imaging examinations performed. Einstein et al noted a 64% reduction in invasive and non-invasive diagnostic tests for heart disease from March 2019 to April 2020 over 909 centers in 108 countries [10]. One year later in the INCAPS COVID-2 survey, Einstein et al noted an almost full recovery to 97% of worldwide diagnostic cardiac imaging procedures and an overall recovery above the baseline of cardiac MRI examinations comparing March 2019 to April 2021 [12]. The recovery rate differed between countries, being above in the US [33] and below the baseline in Italy [34]. Compared to the INCAPS studies, our work adds to the literature more detailed analyses including the indications for cardiac MRI examinations.

Multifactorial causation has been proposed for COVID-19-associated myocarditis. Fairweather et al [21] identified important attributes potentially linking COVID-19 to an elevated risk of myocarditis. For one, the spike protein of SARS-CoV-2 binds angiotensin-converting-enzyme 2 (ACE2) which is expressed in cardiac cells. After initially infecting the lungs (COVID pneumonia), the SARS-CoV-2 virus, inflammatory cytokines, and activated immune cells can travel to the heart. Via the ACE2–spike protein interaction infection of cardiac cells is facilitated. The circulating inflammatory cells and resident immune cells react to the viral load and damage cardiac tissue, causing myocarditis. Similar mechanisms were proposed for vaccination-associated myocarditis where vaccination may boost the immune response in preexisting subclinical cardiac inflammatory conditions [35].

It has been reported that a SARS-CoV-2 infection increases the risk for myocarditis 15- to 16-fold [21, 35, 36] from 9/100,000 to about 150/100,000, with variability depending also on the virus variants. The excess cases for vaccination-associated myocarditis are much lower, being at around 2–10/100,000, and seem to be especially related to mRNA-based vaccines [21, 35].

In accordance with these numbers, we found a relative increase in the MRI examinations performed for myocarditis when comparing pre-pandemic to pandemic data. However, we also found a decrease in the positivity rate for these examinations, meaning that fewer positive cardiac MRI studies for myocarditis were carried out in that period. This may indicate a lower threshold for seeking medical advice for unspecific symptoms during a pandemic with unprecedented media coverage resulting in a lower admission threshold for admission to cardiac MRI by referring physicians. Together this may have led to an increased proportion of cardiac MRI examinations and a reduced positivity rate.

We observed the largest increase in MRI examinations for suspected myocarditis in the first half of 2021, which coincides with the first winter wave of COVID-19 cases in Europe [37], and a comparatively lower number of SARS-CoV-2 infections in 2020 due to initially very strict and wide-spread lockdown and hygiene measures. This steep increase early after the beginning of the pandemic may also partially be caused by a delay in the awareness of, and research on COVID-19-associated myocarditis due to the novelty of the disease. Not all SARS-CoV-2 strains carry the same risk for myocarditis, with the Delta variant emerging in late 2020 [38] associated with a particularly high risk [21]. This may additionally play a role in the steep increase observed in early 2021. Interestingly, our study using the multinational MRCT-registry data indicates that the patterns of referral to cardiac MRI started to return to the pre-pandemic situation, in parallel to the fact that the emergency status has been declared ended by the WHO [32].

The most susceptible population group for COVID-19 and vaccination-associated myocarditis are young males typically between 12 years and 40 years of age [21]. This is in line with the age of the cohort of patients undergoing cardiac MRI for myocarditis in the pre-pandemic time period (predominantly male, below 50 years of age). The reported proportion of male-to-female patients with myocarditis varied widely before the pandemic (60%:40% to 80%:20%) [39]. For COVID-19 and vaccination-associated myocarditis, most studies report a male predominance of 60% to 70% [21]. For patients examined for myocarditis and diagnosed with myocarditis, we found a slight increase in mean age and a slight decrease in the male/female proportion.

Our study is subject to limitations. First, it is a retrospective study based on registry data which may introduce reporting bias. However, this should be true for all examinations and indications and therefore be mitigated to some degree. Second, in this registry no imaging data is available and thus no retrospective image review was possible. Third, no follow-up data is available in the registry to analyze the outcome of patients with suspected myocarditis before and during the pandemic. Fourth, indications for cardiac MRI were not mutually exclusive, which causes an overlap of reported indications, including particularly the indication of known coronary artery disease and viability assessment. This may have skewed the proportions between indications. Fifth, there are inherent fluctuations in the total number of yearly entries in the international registry across countries, which limits the analysis of trends to proportions rather than absolute numbers. Absolute numbers were thus not provided in the current study, but are available from the authors upon reasonable request. Sixth, data in the registry are unevenly distributed between European countries, introducing potential bias and confounding. Seventh, due to the anonymization multiple examinations in the same patients cannot be identified. This however may affect all indications and therefore may be mitigated to some degree. Finally, due to the registry’s inception years before the pandemic, COVID-19 infections and COVID-19 vaccination status are not reported specifically in the registry. Thus, the potential effects of COVID-19 infection vs potential vaccination effects could therefore not be differentiated in this analysis.

In conclusion, we found that the COVID-19 pandemic was associated with a considerable increase in the proportion of cardiac MRI examinations performed for the assessment of myocarditis, while the positivity rate for the diagnosis of myocarditis showed a decrease, which indicates a lower admission threshold for this imaging examination. We also found that during the same period, the proportion of cardiac MRI indications performed for suspected and known CAD decreased. Future studies should evaluate the long-term impact of the COVID-19 pandemic on imaging practices including patient outcome data.

## Supplementary information

Supplementary Material
