# Supplementary material for: Impact of the COVID-19 pandemic on cardiac magnetic resonance imaging practices: insights from the MRCT registry
Source: Eur Radiol. 2025 Feb 19;35(8):4805–13. doi: 10.1007/s00330-025-11464-w (PMC12226639; doi:10.1007/s00330-025-11464-w)
Supplement: Supplementary file 1 — Supplementary Material [file 330_2025_11464_MOESM1_ESM.pdf]

Impact of the COVID-19 pandemic on cardiac magnetic resonance imaging practices: Insights from the MRCT-registry

ELECTRONIC SUPPLEMENTARY MATERIAL

Table S1: List of countries registered in the ESCR registry. Non-European countries in cursive.

**Country**
Argentina
Austria
Bangladesh
Belarus
Belgium
Bulgaria
Canada
Croatia
France
Germany
Greece
Hungary
Iceland
India
Ireland
Italia
Italy
Kazakhstan
Latvia
Lithuania
Luxembourg
Mexico
Netherlands
Poland
Portugal
Republic of Moldova
Romania
Russia
Serbia
Slovakia
Slovenia
Spain
Switzerland
Turkey
Ukraine
United Kingdom
